# Solving the puzzle of quality of life in cancer: integrating causal inference and machine learning for data-driven insights

**DOI:** 10.1186/s12955-024-02274-7

**Published:** 2024-08-07

**Authors:** Hakan Şat Bozcuk, Mustafa Serkan Alemdar

**Affiliations:** Dept. of Medical Oncology, Medical Park Hospital, Antalya, Turkey

**Keywords:** Causal inference, Quality of life, Cancer, Machine learning, Regression analysis, Social functioning, Emotional functioning

## Abstract

**Background:**

Understanding the determinants of global quality of life in cancer patients is crucial for improving their overall well-being. While correlations between various factors and quality of life have been established, the causal relationships remain largely unexplored. This study aimed to identify the causal factors influencing global quality of life in cancer patients and compare them with known correlative factors.

**Methods:**

We conducted a retrospective analysis of European Organization for Research and Treatment of Cancer Quality of Life Questionnaire data, alongside demographic and disease-related features, collected from new cancer patients during their initial visit to an oncology outpatient clinic. Correlations with global quality of life were identified using univariate and multivariate regression analyses. Causal inference analysis was performed using two approaches. First, we employed the Dowhy Python library for causal analysis, incorporating prior information and manual characterization of an acyclic graph. Second, we utilized the Linear Non-Gaussian Acyclic Model (LiNGAM) machine learning algorithm from the Lingam Python library, which automatically generated an acyclic graph without prior information. The significance level was set at *p* < 0.05.

**Results:**

Multivariate analysis of 469 new admissions revealed that disease stage, role functioning, emotional functioning, social functioning, fatigue, pain and diarrhea were linked with global quality of life. The most influential direct causal factors were emotional functioning, social functioning, and physical functioning, while the most influential indirect factors were physical functioning, emotional functioning, and fatigue. Additionally, the most prominent total causal factors were identified as type of cancer (diagnosis), cancer stage, and sex, with total causal effect ratios of -9.47, -4.67, and − 1.48, respectively. The LiNGAM algorithm identified type of cancer (diagnosis), nausea and vomiting and social functioning as significant, with total causal effect ratios of -9.47, -0.42, and 0.42, respectively.

**Conclusions:**

This study identified that causal factors for global quality of life in new cancer patients are distinct from correlative factors. Understanding these causal relationships could provide valuable insights into the complex dynamics of quality of life in cancer patients and guide targeted interventions to improve their well-being.

## Background

In recent decades, while the primary focus of cancer treatment has been on achieving a cure or prolonging survival, there has been a growing recognition of the importance of improving the quality of life (QoL) for patients with cancer [1]. Various scales and questionnaires are used to assess QoL in different cancer types, such as breast cancer, but not all of these tools have been universally validated across all settings and cultures [2, 3]. One of the most extensively studied instruments for cancer patients is the European Organization for Research and Treatment of Cancer Quality of Life Questionnaire (EORTC QLQ-C30) [4, 5]. Although global QoL is a key dimension of this scale, it is not merely the sum of its individual parts, which encompass various dimensions of QoL [6]. Despite our understanding that many disease- or treatment-related factors may influence specific QoL dimensions, the underlying causal factors of global QoL remain largely unexplored.

Although predictive and explanatory models in the field of medicine have predominantly adopted a correlational approach, it is essential to recognize that correlation does not imply causation. Identifying causal factors is crucial for evaluating the impact of interventions, policy changes, or exposure to certain factors on outcomes, particularly in the medical domain [7]. Artificial intelligence (AI) has found widespread applications in medicine for tasks such as diagnosis, research, and prediction, often leveraging correlated data [8]. However, to overcome the limitations of working solely with correlated data, it is imperative to conduct special analyses, such as causal inference. This kind of analysis theoretically enables clinicians and researchers to discern causal factors and evaluate treatment effects using observational data, thereby providing insights into complex networks of relationships [9]. Therefore, gaining a deeper understanding of the causal factors underlying QoL is pivotal for enhancing the overall well-being and QoL of cancer patients.

Machine learning (ML), a critical subset of artificial intelligence (AI), involves the creation of algorithms that can learn from data and make predictions or decisions without explicit programming for specific tasks. This capability allows ML to analyze vast and complex datasets, identifying patterns and making informed predictions that can significantly enhance various domains, including medicine. Integrating ML with causal inference allows researchers and clinicians to not only predict outcomes but also understand the underlying mechanisms driving these outcomes, leading to more effective and targeted interventions.

For this reason, in this study, we sought to explore the causal factors influencing global QoL by analyzing observational data collected from consecutive cancer patients during their initial appointments at a cancer center. By applying analytical methods capable of identifying causal relationships, we aimed to shed light on the intricate web of factors influencing global QoL in cancer patients.

## Methods

### Patient selection and data collection

All patients with cancer who visited a private hospital’s cancer center and were under the care of the same attending physician were provided with the Turkish version of the EORTC QLQ-C30 version 3 questionnaire during their initial appointment at the center [10]. We included quality of life data from all consecutive new patients who consented to complete the questionnaire between March 2018 and April 2021. Additionally, we collected primary demographic and disease-related information.

Quality-of-life dimension scores were computed following the guidelines outlined in the scoring manual. Quality of life dimensions of the EORTC QLQ-C30 questionnaire were QL2; global quality of life, PF2; physical functioning, RF2; role functioning, EF; emotional functioning, CF; cognitive functioning, SF; social functioning, FA; fatigue, NV; nausea and vomiting, PA; pain, DY; dyspnea, SL; insomnia, AP; appetite loss, CO; constipation, DI; diarrhea, and FI; financial difficulties. The study was approved by the ethical committee of the Medical Park Hospital, Antalya, on the 17th of January 2024, with a reference number of 2024/1.

### Evaluation of correlative factors for global quality of life

In order to contrast with the findings from evaluation of causal factors for global quality of life, we first wanted to conduct a non-causal, univariate regression analysis of potential predictor variables for global QoL (QL2). Variables with a p value of 0.10 or less in the univariate analysis were then included in the multivariate regression analysis. Stepwise method was then used in the multivariate analysis to select the significant variables. For diagnostic purposes, we categorized patients with breast cancer versus other cancers or with lung or colorectal cancer versus other cancers. We considered a p value less than 0.05 to indicate statistical significance. The statistical analyses were conducted using SPSS version 21.

### Evaluation of causal factors for global quality of life

To investigate causal inference, we employed two distinct approaches. Firstly, we manually constructed a directed acyclic graph (DAG) using domain knowledge and the results of the preliminary exploratory analysis of the dataset (Expert-driven construction of the DAG). DAGs model the causal relationships among the variables of interest. DAGs are graphical representations where nodes signify variables and directed edges denote causal effects, with the constraint that no cycles exist, ensuring a unidirectional flow of causation. We constructed our DAGs based on domain knowledge and prior empirical evidence to accurately capture the underlying causal mechanisms. These graphs were instrumental in identifying confounding variables, mediators, and potential sources of bias, thus guiding our statistical analysis and ensuring the robustness of our causal inferences. By leveraging DAGs, we aimed to achieve a clear and precise delineation of the causal pathways influencing the outcomes of our study, ultimately enhancing the validity and interpretability of our findings.

To construct DAG, we utilized DoWhy Python library, which is used for evaluating causal inference, to calculate and plot the separate impacts of direct, indirect, and total causal effects [11, 12]. Percentage direct causal strength quantifies the influence of a specific factor (or node) on another within a directed acyclic graph (DAG) in terms of direct causation. It measures the proportion of total direct causal impact exerted by each factor relative to the sum of all direct causal impacts on the target variable. Additionally, the percentage indirect causal effect quantifies the influence of a specific factor on an outcome through one or more intermediary variables within a DAG. It measures the proportion of the total indirect causal impact exerted by each factor relative to the sum of all indirect causal impacts on the target variable. As stated above, to be included in DAG, factors either had to be associated with each other during the exploratory statistical analysis, or had to be linked according to the oncology domain knowledge.

Secondly, we utilized the Lingam Python library to implement a fully automated machine learning approach based on the Linear Non-Gaussian Acyclic Model (LiNGAM) [13]. LiNGAM is a machine learning algorithm used in causal discovery, specifically designed to identify causal structures from observational data. LiNGAM assumes that the data follows a linear relationship and that the errors (or noise) are non-Gaussian. This assumption allows the algorithm to distinguish between cause and effect, which is a significant advantage over other linear methods that might not be able to differentiate between the two. LiNGAM is also used in fields like econometrics, genomics, and various social sciences where understanding the causal relationships between variables is crucial. It operates under the principle that if the data generation process follows a linear non-Gaussian model, then it is possible to identify the causal ordering of the variables and the corresponding causal effects. In our study, LiNGAM allowed us to visualize the LiNGAM adjacency matrix and calculate the total causal effects of various factors [14, 15]. We employed these two approaches (DAG and LiNGAM) basically to more soundly and validly test causal assumptions and relationships that are hidden in our dataset.

These analyses aimed to uncover not only the associational relationships between variables and global quality of life but also the potential causal factors influencing it. By employing the above statistical methods and leveraging both domain knowledge and automated machine learning algorithms, we sought to gain a deeper understanding of the complex interplay of factors shaping the overall quality of life experienced by cancer patients attending the center in the specified time period.

During the preparation of this work, the authors used Chat GPT 3.5 to improve readability. After using this tool, the authors reviewed and edited the content.

## Results

### General results

**Fig. 1:**
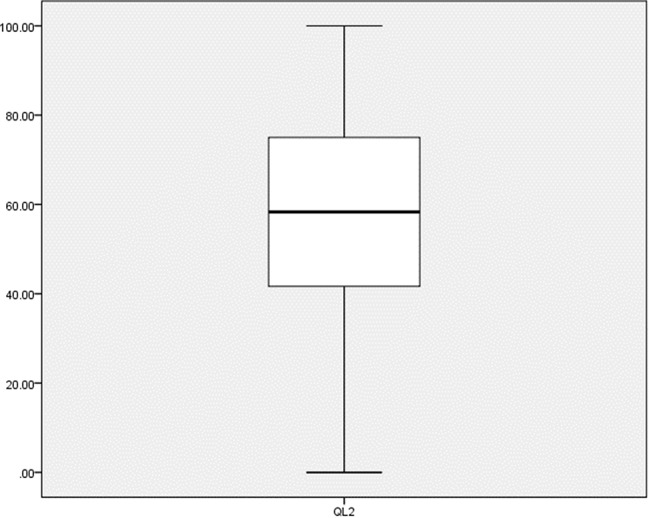
Distribution of Global Quality of Life (QL2) scores. Global quality of life distribution. QL2; Global quality of life. The Y-axis shows the scores on a scale ranging from 0 to 100

A total of 469 patients were recruited, with a median age of 56 years and a 63% female distribution. Breast cancer was the most common diagnosis and was noted in 40% of patients. The other cancers category (37%) included gastric cancer (4%), prostate cancer (4%), testis cancer (4%), skin cancer (2%) and ovarian cancer (2%) as the most prominent subtypes, and remaining various other cancer types (21%). The patient demographics and details can be found in Table 1. The median value for QL2 was 58 on a scale of 100. The distribution of QL2 is presented in Fig. 1.

**Table 1:**
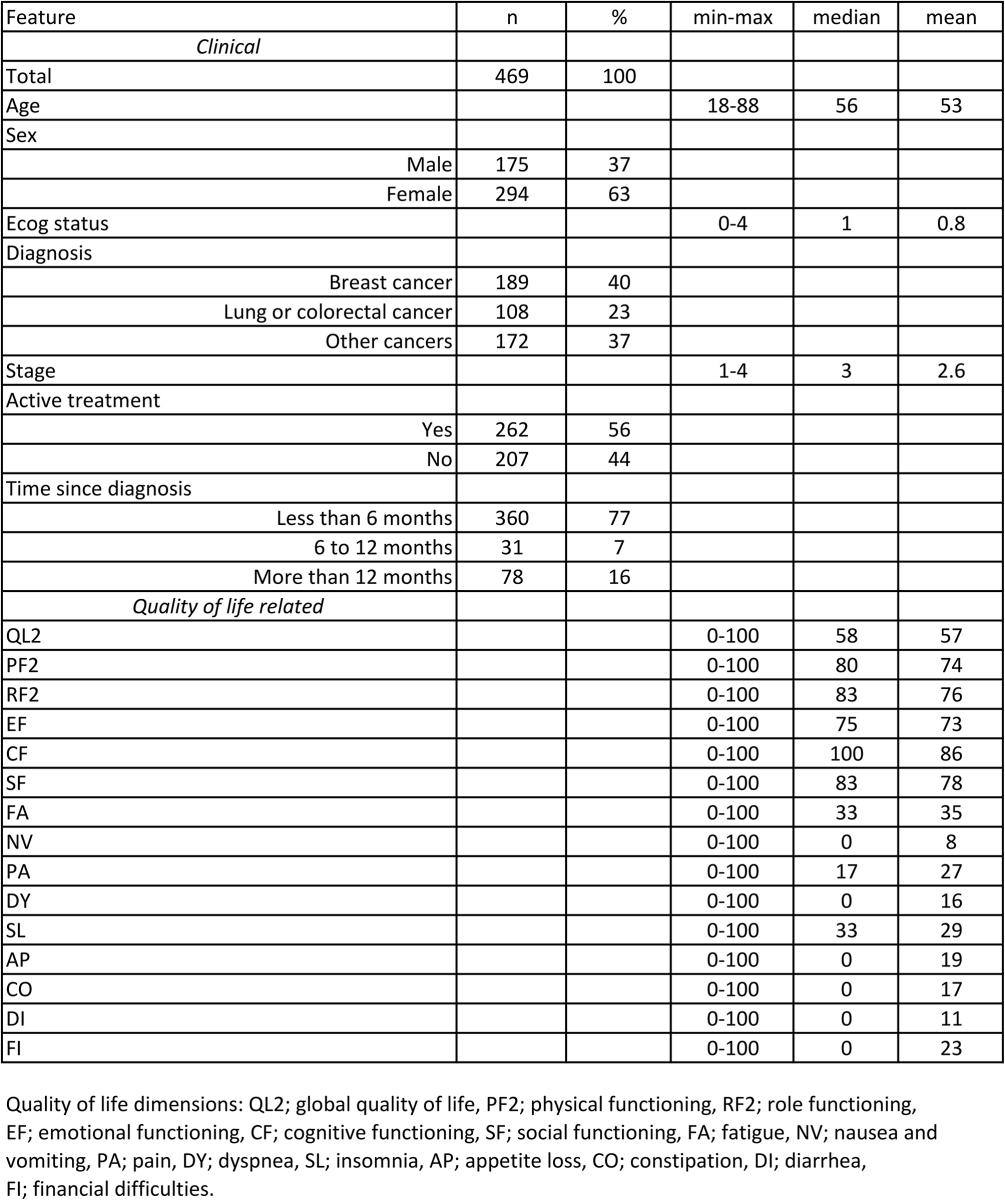
Demographics and quality of life data

### Associates of global quality of life

According to the univariate analysis, all quality-of-life dimensions individually and diagnosis (lung or colorectal vs. other cancers) and disease stage were associated with the QL2 score. The feature with the highest t value was fatigue (FA) scores, with a beta of -0.61, t = -16.60, and *P* < 0.001. According to the multivariate analyses, 7 factors were significant, namely, disease stage, role functioning (RF2), emotional functioning (EF), social functioning (SF), fatigue (FA), pain (PA) and diarrhea (DI), and the factor with the highest t value was SF, with a beta of 0.18, t = 3.71, and *P* < 0.001. The details of the regression analyses can be found in Table 2.

**Table 2:**
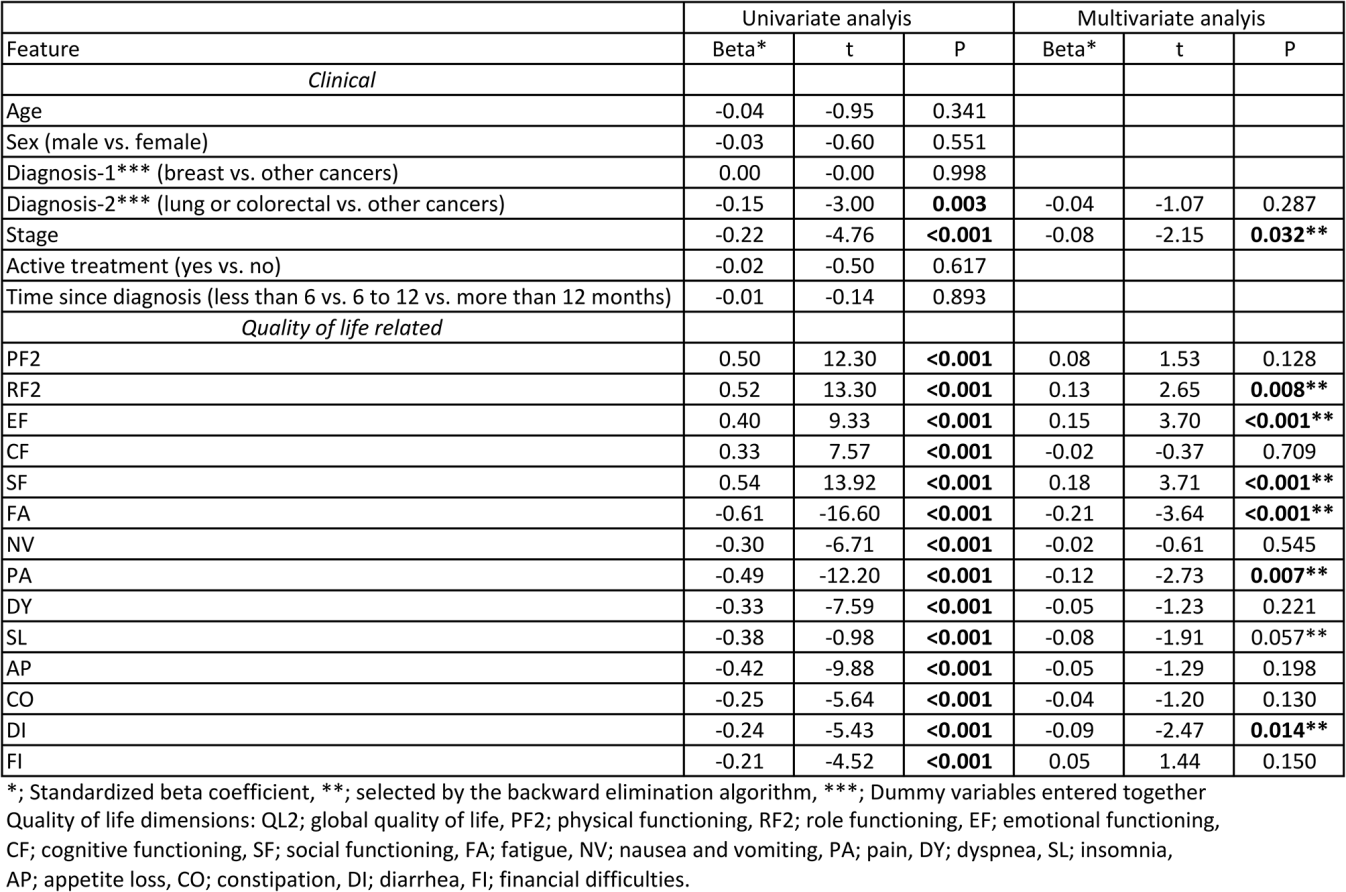
Associates of global quality of life

### Causal factors for global quality of life

#### Expert-driven construction of the DAG

**Fig. 2:**
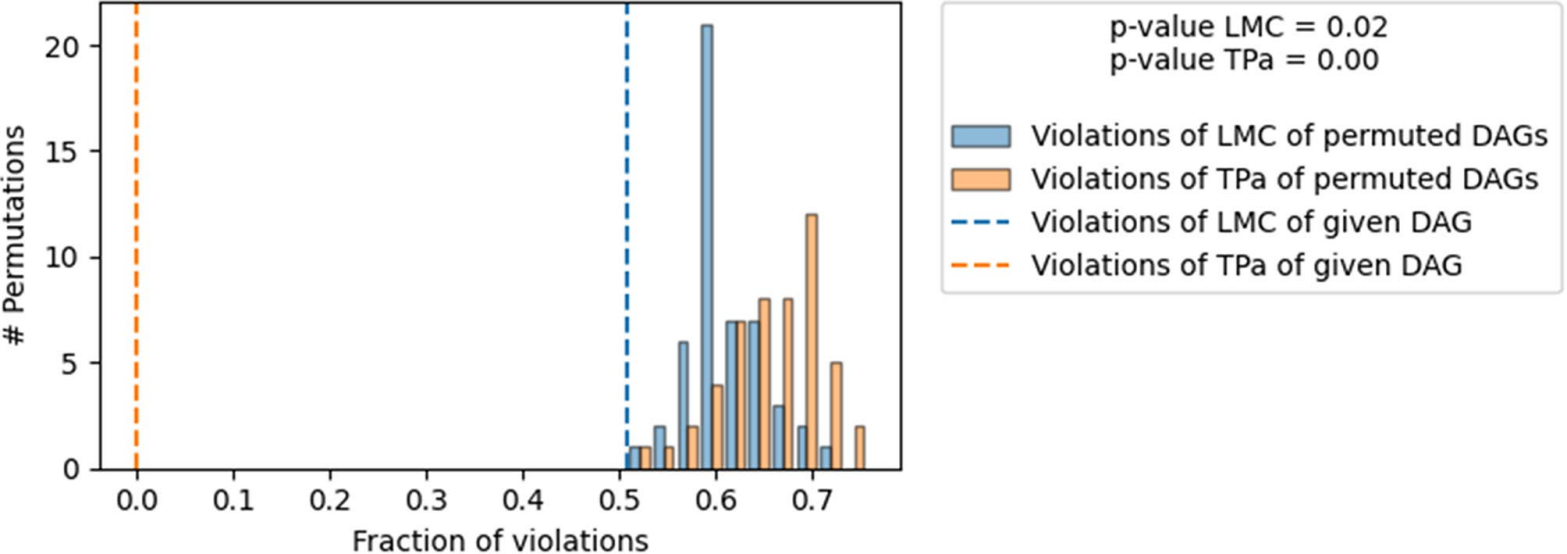
Evaluation of directed acyclic graph (DAG). Directed acyclic graph structure. Directed acyclic graph (DAG) network of causal relationships among variables. Quality of life dimensions: NV, nausea and vomiting; SL, insomnia; AP, appetite loss; CO, constipation; DI, diarrhea; FI, financial difficulties; PF2, physical functioning; FA, fatigue; PA, pain; DY, dyspnea; RF2, role functioning; EF, emotional functioning; CF, cognitive functioning; QL2, global quality of life; SF, social functioning. Time_after_dx; time elapsed after diagnosis, diagx2_2d; type of cancer diagnosis (lung or colorectal cancer versus other cancers), active_treatment; whether the patient is on active treatment, stage; TNM stage of cancer

With expert-driven construction of the DAG, the resultant model was found to provide explanatory value among the possible permuted DAGs, as shown by a *P* value LMC = 0.02 and a *P* value TPa < 0.01, which are both statistically significant. The plot for causal model evaluation is given in Fig. 2. The plot and the accompanying tests show that the given DAG structure has a lower fraction of violations for both the Local Markov Condition and Transitive Pairing compared to randomly permuted DAGs. The low p-values suggest that these results are statistically significant, indicating that the DAG is a valid and explanatory model for the underlying causal relationships in the data.

**Fig. 3:**
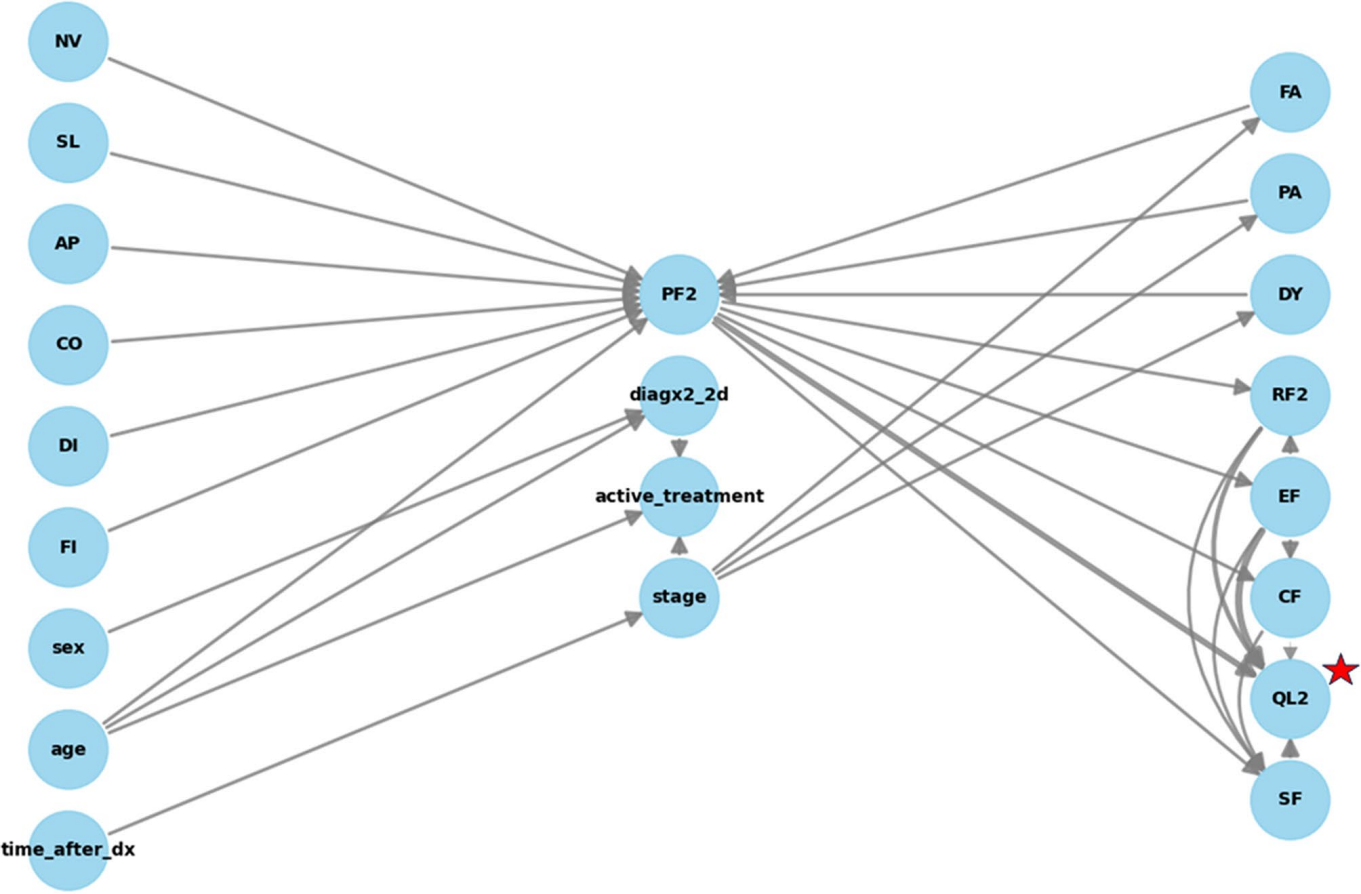
Causal graph for global quality of life. Directed acyclic graph model evaluation. DAG: Directed acyclic graph. Two tests show that the DAG structure is explanatory

The plotted DAG with the network of causal relationships among the causal factors and QL2 is detailed and can be visualized in Fig. 3.

The DAG structure has some underlying patterns, although not all causal effects are of large amplitude, for example, a number of clinical factors and quality of life domains on the left column in Fig. 3 appear to effect PF2; physical functioning, among others, and PF2 possesses direct and indirect causal effects (through EF; emotional functioning and CF; cognitive functioning) on QL2; global quality of life.

Among the direct causal factors, the most influential was emotional functioning, with a percentage direct causal strength of 38%. When indirect causal factors were considered, physical functioning was at the top, with a percentage indirect causal strength of 30%. Finally, among the total causal factors, diagnosis (lung or colorectal cancer versus other cancers) was the most influential causal factor, with a causal effect ratio of -9.47, other prominent total causal factors were stage and sex. DAG did not show valid paths for some of the causal associations between various factors, including diagnosis, and QL2, which is the main outcome of interest. In causal analysis, unobserved variables (also called latent variables) can play a crucial role in the causal relationships between observed variables, and as such, the plotted DAG we formulated may conceal some of these latent variables. The plots for causal factors found by the DAG structure are not separately given for the purpose of brevity.

#### LiNGAM machine learning causal algorithm

**Fig. 4:**
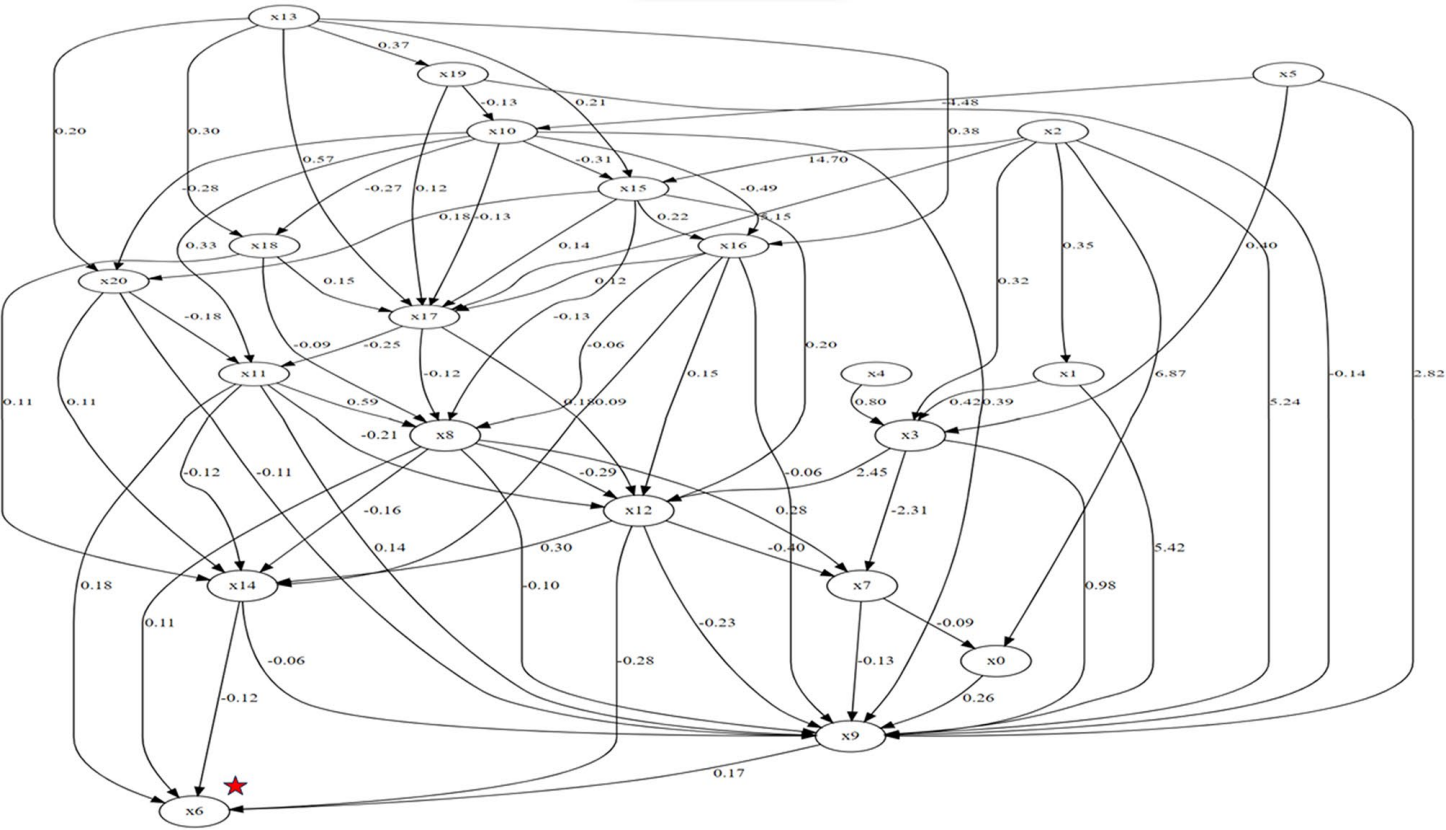
LiNGAM adjacency matrix of causal factors. Linear Non-Gaussian Acyclic Model (LiNGAM) adjacency matrix. Network of complex causal relationships among variables in the study. x0; age, x1; sex, x2; type of cancer diagnosis (lung or colorectal cancer versus other cancers), x3; TNM stage of cancer, x4; whether the patient is on active treatment, x5; time elapsed after diagnosis, x6; global quality of life, x7; physical functioning, x8; role functioning, x9; emotional functioning, x10; cognitive functioning, x11; social functioning, x12; fatigue, x13; nausea and vomiting, x14; pain, x15; dyspnea, x16; insomnia, x17; appetite, x18; constipation, x19; diarrhea, x20; financial difficulties. Each node in the plot represents a variable, and the directed edges between nodes indicate the direction of causality. LiNGAM aims to identify the causal ordering of variables and their causal effects, so the plot will show the inferred causal structure based on the analysis of the data

With the Lingam algorithm, the adjacency matrix showed the network and direction of connections among the causal factors themselves and the causal factors directly and indirectly affecting QL2 and QL2 itself. There were 5 direct causal connections between various causal factors and QL2, namely, from EF to QL2, from FA to QL2, from PA to QL2, from RF2 to QL2, and from SF to QL2. The adjacency matrix is presented in Fig. 4. Interestingly, the causal effect direction in Fig. 4 between diagnosis and sex is from diagnosis to sex, whereas, we would expect the contrary, as difference in gender would be causing different cancers, such as female gender is expected to have more breast cancers, compared to the male gender. Here, apparently, the model wrongly guesses the direction of causal effect. In some cases, the relationship might be bidirectional or more complex than a simple cause-effect direction. LiNGAM might struggle with such relationships, leading to incorrect causal directions, which we think is the case here.

**Fig. 5:**
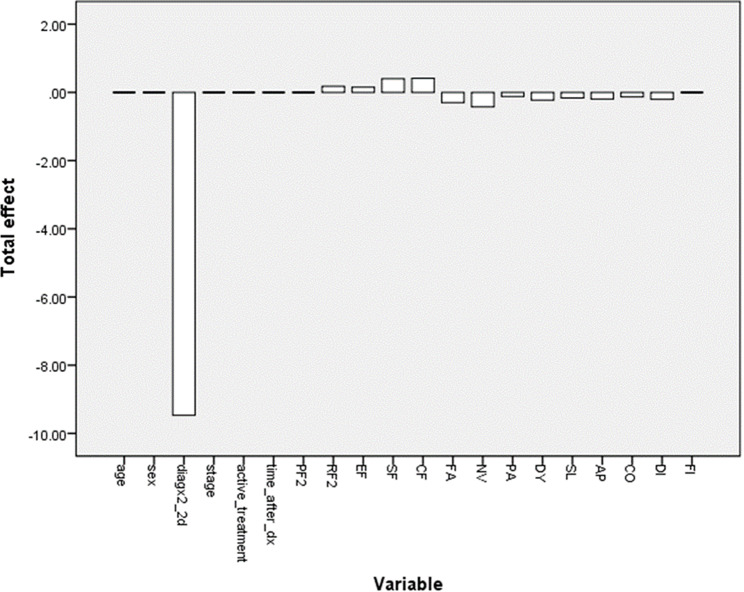
Total causal effects of LiNGAM. Linear Non-Gaussian Acyclic Model (LiNGAM) total causal effect scores. Abbreviations: diagx2_2d: lung or colorectal cancer, or other cancers, stage: cancer TNM stage at admission, active_treatment: receipt of chemotherapy or other systemic treatments, surgery or radiotherapy at the time of admission, time_after_dx: time elapsed since diagnosis at the time of admission, PF2: physical functioning, RF2: role functioning, EF: emotional functioning, SF: social functioning, CF: cognitive functioning, FA: fatigue, NV: nausea and vomiting, PA: pain, DY: dyspnea, SL: insomnia, AP: appetite loss, CO: constipation, DI: diarrhea, FI: financial difficulties

**Fig. 6:**
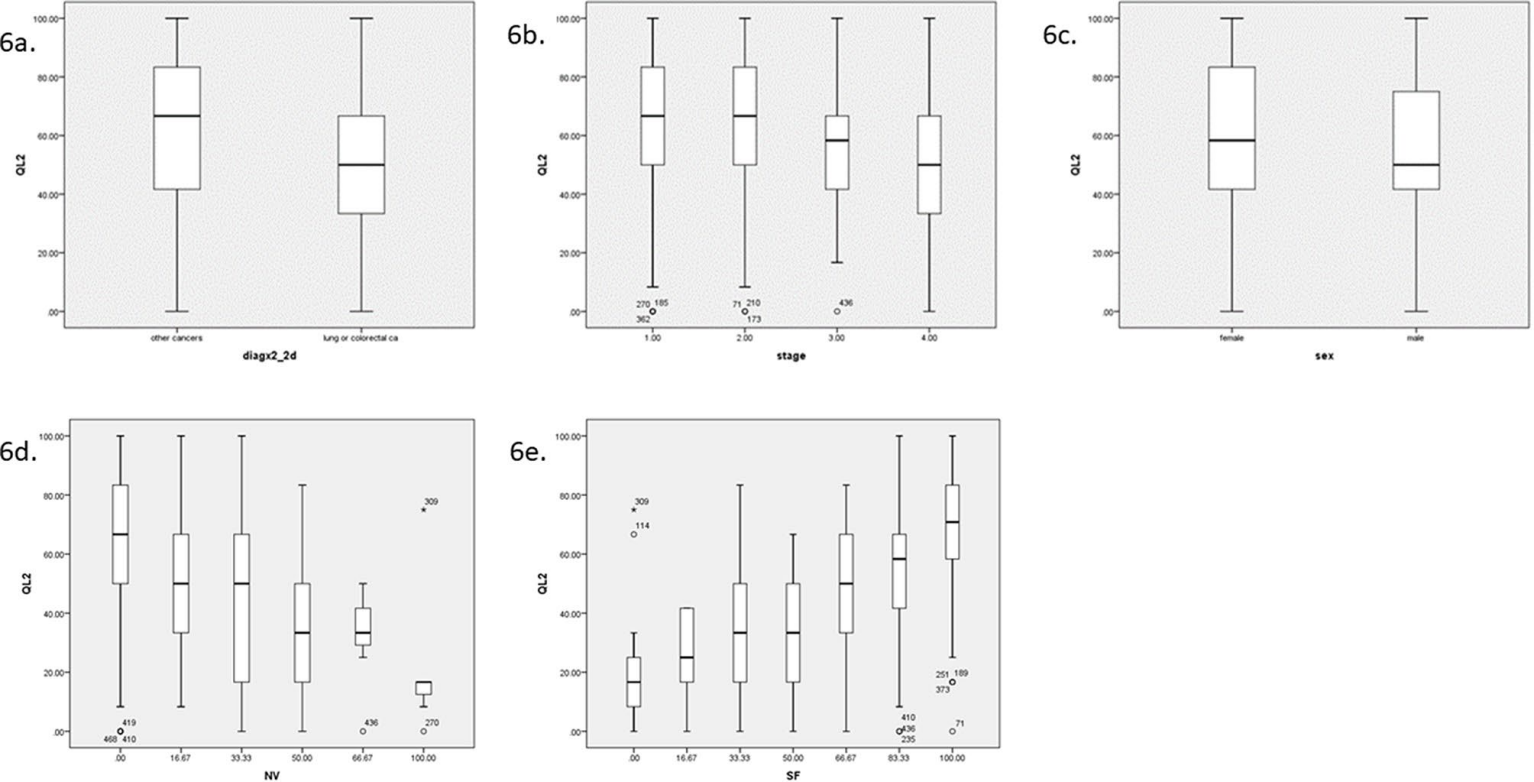
Main total causal effects and global quality of life. Main total causal influences and global quality of life. Variables with the greatest total causal effects on global quality of life, as demonstrated in the Directed Acyclic Graph (DAG) and Linear Non-Gaussian Acyclic Models (LiNGAM). Global quality of life with reference to **6a**: diagx2_2d; type of cancer diagnosis (lung or colorectal cancer versus other cancers), **6b**: disease stage, **6c**: sex, **6d**: NV; nausea and vomiting, **6e**: SF; social functioning. Figure 6 includes diagnosis, stage and sex from DAG, and diagnosis, NV and SF from LiNGAM

When total causal effects were considered, the most influential causal factor for QL2 was again the diagnosis (lung or colorectal cancer versus other cancers), as in the case with expert driven construction of the DAG. Figure 5 shows the causal effects of other factors on QL2. The main causal factors affecting QL2 revealed by manual DAG construction and the LiNGAM algorithm are detailed in Fig. 6, which shows the causal effects of diagnosis, stage, sex, nausea vomiting (NV), and SF on QL2.

## Discussion

We have demonstrated, for the first time, that the type of cancer diagnosis, specifically the presence of lung or colorectal cancer versus other cancers, exhibits a causal relationship with global quality of life as shown by 2 separate methods of causal analysis. While some previous studies have indicated an association between cancer type and various quality of life scores, our analysis using machine learning and Bayesian inference techniques revealed that this relationship is not purely correlative but has a causal component [16]. In contrast, some authors have not shown a difference in global quality of life with respect to cancer type [17]. Interestingly, the most influential causal factors for QL2 identified in this study differ from the strongest associates of QL2. Although the type of cancer diagnosis emerged as the most influential causal factor for QL2 in both the manual approach and the LiNGAM algorithm, it did not reach statistical significance in the multivariate analysis conducted using classical regression analysis. Similarly, while social functioning (SF) was the strongest associate of QL2 in the regression analysis, it did not emerge as the most influential causal factor in our causal analyses. This disparity underscores some unique insights that causal analysis could offer, revealing complex causal relationships that might not be captured by traditional associative or correlative approaches, such as regression analysis.

The DAG we manually coded did not include a visible path between the type of cancer diagnosis and QL2, but the total causal effect of the type of cancer diagnosis could be quantified algorithmically. However, looking at the LiNGAM adjacency matrix, we see that there are various possible paths between the type of cancer diagnosis and QL2, and thus, in that fashion, the LiNGAM adjacency matrix captured the complex relationships in our study better than the DAG. A possible explanation for the discrepancy between these 2 separate approaches (Expert-driven construction of the DAG versus LiNGAM) in our study is that the manually coded DAG failed to capture some of the important variables that were visible in the LiNGAM adjacency matrix. In other words, these variables remained latent for some of the relationships in the DAG structure, making LiNGAM, a machine learning model, more explanatory for our purpose.

The use of DAG and LiNGAM approaches in causal analysis offers several strengths and limitations. DAGs provide a clear and intuitive graphical representation of causal relationships, aiding in the identification of confounders, mediators, and potential sources of bias [18]. They facilitate the understanding of complex causal structures and the formulation of testable hypotheses. LiNGAM extends this by enabling the discovery of causal ordering and effects from observational data under the assumptions of linearity and non-Gaussian errors, offering a robust method for causal inference when these conditions are met. However, both approaches have limitations. DAGs rely heavily on domain knowledge for accurate construction, and their effectiveness is constrained by the correctness and completeness of this prior knowledge. LiNGAM’s assumptions of linearity and non-Gaussianity may not always hold in real-world data, potentially limiting its applicability and accuracy. Additionally, LiNGAM can be sensitive to sample size and may struggle with high-dimensional data. Despite these limitations, the combined use of DAGs and LiNGAM provided a powerful toolkit for causal discovery and analysis for our study.

If the type of cancer directly influences global quality of life at the time of presentation, several factors may drive this phenomenon. Previous studies, including ours, have assessed quality of life across different cancer populations and have identified differences in various dimensions of quality of life; those cancer populations included metastatic or nonmetastatic cases, men or women, those with different categories of weight loss, performance status, weight loss, time from last treatment, and emotional and functional well-being [16, 19–21]. Specifically, the literature shows differences in the QL2 score between lung cancer patients and patients with other types of cancer [22]. Likewise, the quality-of-life score is also low in patients with colon cancer [23]. These differences are likely attributed to the distinct biological profiles of various cancers, with lung cancer often presenting at more advanced stages and having a poorer prognosis, especially in patients with stage 4 disease. Causal factors alter the magnitude of the affected variable, leading to a difference in its magnitude. This aligns with the basic concept of causality, where one variable (the cause) influences another variable (the effect). Thus, from this perspective, type of cancer causally effects and leads to difference in global quality of life in cancer patients. Additionally, the stigma associated with different types of cancer and perceptions of their curability may impact patients’ experiences [24, 25]. For instance, a lung cancer patient may perceive a more unfavorable outlook than a patient with breast cancer. Thus, not only the biological and symptomatic aspects of the disease but also the patient’s perception and societal view of the disease may influence the QL2 score. Further research focusing on the perception dimension and its relationship with quality-of-life dimensions across various cancers could provide valuable insights in this direction.

Apart from type of cancer, which has a causal effect on global quality of life, stage also emerged as another causal factor. Additionally, functional dimensions of QoL had positive causal effects, whereas symptom scales had negative causal effects on QoL2. Although there is some evidence that there is no association between stage or functional dimensions of QoL and QoL2, some available evidence suggests that some domains of QoL may be marginally different with respect to the disease stage [17, 21].

While our study provides important insights, it is not without limitations. Despite including data from 469 patients, a larger sample size, potentially in the range of thousands of cases, would be preferable for elucidating more intricate causal relationships and accurately assessing their strength. Additionally, obtaining more comprehensive data on social security, financial status, treatment details, and patients’ perceptions of their disease status would enable a more thorough examination of the causal effects of these factors in addition to the other variables considered in this study. Lastly, the validity of LiNGAM has been proven only for continuous variables; thus, our use of LiNGAM, which includes categorical or discrete data, is experimental.

The rise of AI and machine learning techniques is reshaping medical practice [26]. These technologies, including computer vision, natural language processing, and various machine learning algorithms, have been increasingly utilized for predicting outcomes and prognosis in recent years. Causal inference, whether through probabilistic, statistical, or machine learning-oriented algorithms, addresses the question of “Why did this happen?” rather than “What features or factors are associated with this outcome?” [27, 28]. This approach is expected to influence medical policies and interventions by providing deeper insights into causal relationships. Furthermore, causal inference techniques, such as causal survival analysis, are gaining traction in other research areas and are anticipated to play a more prominent role in medical research in the future [29]. The findings from our study lend support to this trend.

In summary, our study highlights the causal effect of cancer type, among other factors, on changes in QL2 scores in cancer patients. Importantly, this causal effect was not evident in classical regression analysis. The causal inference methodologies employed in our study have the potential to inform policies and interventions aimed at improving quality of life in cancer patients and, more broadly, to address numerous causal questions in oncology and medicine.

## Conclusions

We found that cancer type is the primary causal factor for global quality of life in cancer patients. Additionally, our causal analyses revealed different factors compared to those identified in the regression analyses.

## Data Availability

The datasets used and/or analyzed during the current study are available from the corresponding author on reasonable request.

## Abbreviations

LiNGAM: Linear Non-Gaussian Acyclic Model
QoL: Quality of life
EORTC QLQ-C30: European Organization for Research and Treatment of Cancer Quality of Life Questionnaire
AI: Artificial intelligence
QL2: global QoL
PF2: Physical functioning
RF2: Role functioning
EF: Emotional functioning
SF: Social functioning
CF: Cognitive functioning
FA: Fatigue
NV: Nausea and vomiting
PA: Pain
DY: Dyspnea
SL: Insomnia
AP: Appetite loss
CO: Constipation
DI: Diarrhea
FI: Financial difficulties
DAG: Directed acyclic graph
Time_after_dx: Time elapsed after diagnosis
Diagx2_2d: type of cancer diagnosis (lung or colorectal cancer versus other cancers)
